# Olfactory training with *Aromastics*: olfactory and cognitive effects

**DOI:** 10.1007/s00405-021-06810-9

**Published:** 2021-04-16

**Authors:** Anna Oleszkiewicz, Laura Bottesi, Michal Pieniak, Shuji Fujita, Nadejda Krasteva, Gabriele Nelles, Thomas Hummel

**Affiliations:** 1 Smell & Taste Clinic, Department of Otorhinolaryngology, TU Dresden, Fetscherstrasse 74, 01307 Dresden, Germany; 2 Institute of Psychology, University of Wroclaw, ul. Dawida 1, 50-527, Wroclaw, Poland; 3 Sony Corporation, New Business & Technology Development Group, 1-7-1 Konan Minato-ku, Tokyo, 108-0075 Japan; 4 Sony Europe B.V., RD Center Stuttgart Laboratory 2, Hedelfingerstr. 61, 70327 Stuttgart, Germany

**Keywords:** Olfaction, Olfactory rehabilitation, Smell, Olfaction disorders, Olfactory training

## Abstract

**Purpose:**

The olfactory system can be successfully rehabilitated with regular, intermittent stimulation during multiple daily exposures to selected sets of odors, i.e., olfactory training (OT). OT has been repeatedly shown to be an effective tool of olfactory performance enhancement. Recent advancements in studies on OT suggest that its beneficial effects exceed olfaction and extend to specific cognitive tasks. So far, studies on OT provided compelling evidence for its effectiveness, but there is still a need to search for an optimal OT protocol. The present study examined whether increased frequency of OT leads to better outcomes in both olfactory and cognitive domains.

**Method:**

Fifty-five subjects (28 females; *M*_age_ = 58.2 ± 11.3 years; 26 patients with impaired olfaction) were randomly assigned to a standard (twice a day) or intense (four times a day) OT. Olfactory and cognitive measurements were taken before and after OT.

**Results:**

OT performed twice a day was more effective in supporting olfactory rehabilitation and interventions targeted to verbal semantic fluency than OT performed four times a day, even more so in subjects with lower baseline scores.

**Conclusions:**

OT is effective in supporting olfactory rehabilitation and interventions targeted to verbal semantic fluency. However, it may be prone to a ceiling effect, being efficient in subjects presenting with lower baseline olfactory performance and lower verbal semantic fluency.

**Supplementary Information:**

The online version contains supplementary material available at 10.1007/s00405-021-06810-9.

## Introduction

Olfactory training (OT) is an innovative, non-invasive intervention targeted to rehabilitation of olfactory dysfunction. Cumulative evidence suggests its effectiveness in the treatment of patients with olfactory loss due to various reasons [1–10]. OT is also helpful in inhibiting olfactory decline related to aging [11, 12]. Neural changes following OT have been reported for peripheral [13] and central parts of the olfactory system [14–16]. Based on the observed changes in messenger ribonucleic acid (mRNA), and protein expression, recent studies suggest that one mechanism explaining the accelerated olfactory system recovery as the consequence of OT relates to an initial stimulation of olfactory receptors followed by neurogenesis or enhanced synaptogenesis wherein olfactory ensheathing cells play an important role [17].

After the discovery that smell loss is a specific symptom of COVID-19 [18–23], olfactory rehabilitation methods received more scientific attention. Although proven to be successful, OT still needs standardization of its protocol. One of the urgent questions is whether more intense training, increasing activation of olfactory receptor neurons (ONRs), can lead to better clinical outcomes. To date, attempts to verify this hypothesis yielded mixed results: OT with multiple set of different odors was reported more efficient in patients with post-infectious olfactory loss [8] while another study showed that using multi-compound mixtures of odors did not significantly boost OT effects [2]. Yet, increasing activation of ORNs can be accomplished in another way—by increasing the frequency of odor exposures per day during OT.

The aim of this study was to test the hypothesis that the increased frequency of OT yields more pronounced effects. The study included both patients and healthy controls who trained with either standard intensity, i.e., sniffing odors twice a day, or an intense sniffing four times a day. We hypothesized that more frequent olfactory stimulation would be associated with improvement of olfactory [10], cognitive [12], and emotional [24–26] functions in our subjects.

## Methods

### Participants

We determined sample size by utilizing G*Power software [27]. Within the repeated-measures design with between-within group interactions (described in detail in “Statistical approach” section), to obtain power of 0.80 with alpha level set to 0.05 to detect moderate effects of *f* = 0.25, the projected sample size was at least 48 subjects. Patients were referred from general practitioners and ENT specialists, while control sample was recruited by the means of personal contact and fliers distributed at the University clinic. Inclusion criteria for patients were: TDI score below 30.75 points [28] and idiopathic or post-infectious or post-traumatic olfactory loss. For healthy controls, exclusion criteria were: Sniffin’ Sticks score below 30.75 points, regular smoking, pregnancy, and acute or chronic sinonasal diseases and other diseases likely to impede the sense of smell [29]. Due to the possibility of dropouts in our sample, 65 subjects were invited to participate. Of those, 10 did not complete the study procedure (i.e., did not show up for the post-training measurement). Excluded subjects were not different from those who remained in the study in terms of sex *χ*^2^(1) = 0.003, *p* = 0.96, age, *t*(63) = 0.92, *p* = 0.36, but their sense of smell, quantified with the total Sniffin’ Sticks score at study entry, was significantly worse (*M* = 20.9 ± 9.0) than those subjects who remained in the study (*M* = 27.8 ± 9.8). Fifty-five participants completed the two measurements (before and after OT). The sample comprised 28 females and 27 males in age ranging from 32 to 85 years (*M* = 58.2 ± 11.3 years). Sample characteristics are presented in Table 1. Twenty-six participants were classified as anosmic or hyposmic (9 with idiopathic olfactory loss; 13 with post-infectious olfactory loss, and 4 with post-traumatic olfactory loss) and 29 participants were classified as normosmic (classification criterion is described in “Procedure” section) [28]. Thirty-two participants performed a standard training regimen (two training sessions per day) and 23 participants performed an intense training regimen (four training sessions per day).

**Table 1:** Descriptive statistics for subjects’ sex, age, and olfactory loss duration

	*N*	Females	Mean age in years (SD)	Mean duration of olfactory loss in months (SD)
Healthy	29	14	57.4 (11.6)	–
Patients	26	14	59.2 (11.2)	29.2 (25.5)
With idiopathic olfactory loss	9	4	58.6 (10.7)	37.9 (33.7)
With post-infectious olfactory loss	13	7	61.1 (12.8)	22 (12.7)
With post-traumatic olfactory loss	4	3	54.5 (5.8)	35 (37.7)

### Procedure

Subjects were tested twice—before and after OT intervention. Before inclusion in the study, a standardized medical interview was pursued to collect information about the factors that could potentially undermine olfactory abilities such as diabetes, smoking, or current infections [29]. All patients received nasal endoscopy to exclude sinonasal causes of olfactory dysfunction, e.g., chronic rhinosinusitis with or without polyposis.

Subjects were asked to refrain from smoking or eating 1 h prior to the testing session and to avoid wearing strong perfumes on the day of testing. Subjects were tested individually in a well-ventilated room. Based on the interview and the results of the Sniffin’ Sticks test for olfactory function, subjects were categorized as hyposmic or anosmic patients (scores =  < 30.5 points) or healthy controls (scores > 30.5 points) [28]. Baseline measurements were taken during the first meeting, including tests for (1) olfactory function: the Sniffin’ Sticks test battery with three subtests for olfactory threshold, discrimination, and identification [30]; (2) retronasal olfaction using 20 selected grocery-available products [30] (3) individual significance of olfaction measured with eighteen statements relating to the use of odors in everyday life [31]; (4) Montreal Cognitive Assessment (MoCA) test for screening mild cognitive impairment (MCI) with the maximum score of 30 points and 26 points being a cut-off for MCI [32]; (5) Controlled Oral Word Association Test (COWAT) measuring spontaneous production of words [33]; (6) verbal semantic fluency task wherein subjects were asked to name as many supermarket-available products as possible within 60 s; (7) Beck Depression Inventory (BDI) comprising 21 items scored from 0 to 3 points, the higher the result the more intense depressive symptoms [34] and (7) Positive and Negative Affect Schedule (PANAS) comprising 10 items to estimate experience of positive and negative effects [35].

Each participant was equipped with an electrical odor dispenser (cylindrical shape, height 8 cm, diameter 2.3 cm [Aromastic; Sony, Tokyo, Japan]) which allowed to distribute 5 odors (initially subjects used: grapefruit, lavender, lemon grass, ylang-ylang, peppermint). Odors were changed for each subject after approximately 3 months OT period (odors were changed to: menthol, thyme, tangerine, green tea, and bergamot). The change of odors was also used to re-instruct the participants in terms of the OT and to reinforce the motivation to perform the procedures.

The choice of odors was guided by (1) pleasantness of the odors, (2) presence of slight trigeminal activation in some of the odors, e.g., peppermint, (3) evaporation characteristics, so that the odors would last for the duration of the experiment, (4) technical issues mostly in terms of compatibility with the odor cartridge, (5) availability, and (6) inspiration from the previous studies [2, 3, 5]. When pushing a button, a quantum (approximately 4 ml odorized air) of the selected odor were released over approximately 1 s using a silent piezo-based air pump; different odors were selected manually by turning a wheel on top of the odor dispenser. Subjects were instructed to sniff each odor for approximately 30 s, by pushing the button repeatedly. They were randomly assigned to one of the two experimental conditions according to the generated numbers they received when entering the study. They either trained twice (standard OT regimen) or four times a day (intense OT regimen). Additionally, they were asked to perform training before or at least 30 min after the meal at intervals of 12 or 6 h, respectively. Subjects were also told that OT should be performed in quiet, odorless places. The study was concluded after reaching the estimated sample size. All subjects who completed the training and made post-training appointment were included. For two patients (1 with post-viral olfactory loss and 1 post-traumatic olfactory loss), we were not able to acquire olfactory performance measurements, so these two subjects were excluded from the models concerning the Sniffin’ Sticks scores.

### Statistical approach

All statistical analyses were performed with SPSS software. We examined potential differences in the duration of OT and age between patient and control groups and between OT regimen groups by the means of independent sample *t* tests. Furthermore, we tested a series of repeated-measures analysis of variance (rm-ANOVA) models. The measurement time point (pre-training vs post-training) was the within subject variable, and group (patients vs. healthy controls) and training regimen (standard vs. intense) were included as the between subject variables. Duration of the training and participant’s age were included as covariates. The same models were tested for the following dependent variables (scores): olfactory threshold, olfactory discrimination, olfactory identification, retronasal olfaction, individual significance of olfaction, MoCA, COWAT, verbal semantic fluency, BDI, and PANAS. Our main interest was in the interaction effects between the time point measurement (pre-training vs. post-training) and group (patients vs. controls), the time point measurement (pre-training vs. post-training) and training regimen (standards vs intense) as well as in the three-way interaction between these factors. In the section “Results”, we report significant interaction effects of interest, all statistical coefficients for the full models are included in Supplementary file 1: Table 1. All estimated marginal means are included in Supplementary File 2: Table 2. A Bonferroni correction was applied all post hoc analyses. Furthermore, we have examined the relationship between changes in the measures olfactory, cognitive and emotional functions, duration of OT, and subjects’ age with Pearson’s *r* correlation. To compare the fraction of subjects who exhibited clinically significant improvement of olfactory performance with regard to the cause of olfactory loss (control vs idiopathic vs post-infectious vs post-traumatic), we used *χ*^2^ distribution.

## Results

The duration of OT ranged from 108 to 340 days (*M* = 208.6, SD = 64.3 days). It did not differ between patients and healthy controls, *t*(53) = 1.43, *p* = 0.16 [− 9.88; 59.12] or between the groups training twice or four times a day, *t*(53) = − 0.39, *p* = 0.70 [− 42.44; 28.63]. There was also no significant age difference between patients and healthy controls, *t*(53) = − 0.59, *p* = 0.56 [− 7.98; 4.35] or between the groups training twice or four times a day, *t*(53) = − 0.42, *p* = 0.68 [− 7.82; 5.2].

### Olfactory threshold

**Fig. 1:**
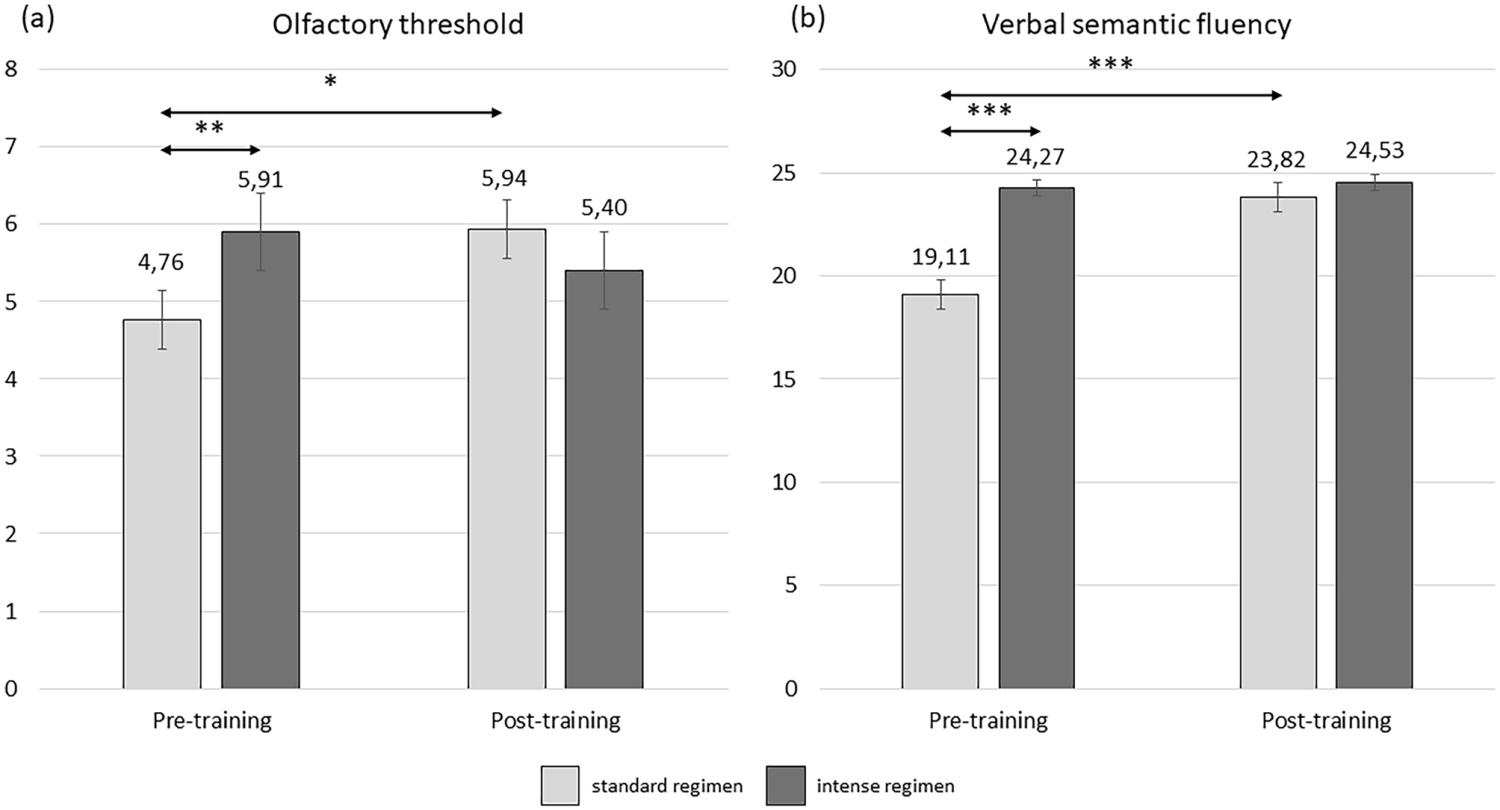
Interaction effects between OT regimen and timepoint measurement for olfactory sensitivity (**a**) and verbal semantic fluency (**b**). *** *p*<.001; ** *p*<.01; * *p*<.05

The group performing OT twice a day had higher olfactory threshold scores after the training in comparison to the baseline measurement (*p* = 0.009). Moreover, during the baseline measurement participants performing OT twice a day had lower threshold scores than participants performing OT four times a day (*p* = 0.041). This interaction effect between time point measurement and training regimen (*F*(1,47) = 5.97, *p* = 0.018, *η*^2^_*p*_ = 0.113) is depicted in Fig. 1a. Additionally, the duration of OT was a significant covariate (*F*(1,47) = 13.88, *p* < 0.001), suggesting that the longer the training, the smaller the increase in olfactory threshold.

### Verbal semantic fluency

The group performing OT twice a day exhibited a significant improvement between the measurements (*p* < 0.001) as opposed to the group training four times a day (*p* = 0.74). Additionally, the group performing OT twice a day showed lower baseline verbal fluency than the group performing OT four times a day (*p* < 0.001). This interaction between the measurement point and the training regimen (*F* = 18.40, *df* = 1,48, *p* =  < 0.001, *η*^2^_*p*_ = 0.277) is depicted in Fig. 1b.

Intercorrelations between the changes (Δ) in olfactory, cognitive and emotional domains, duration of OT, and subjects’ age revealed that age was significantly positively related with increase in positive affect as a function of OT (*r* = 0.29, *p* = 0.03). The increase in MoCA score was negatively related with duration of OT (*r* = − 0.36, *p* = 0.01), suggesting that the prolongation OT duration could inhibit the beneficiary effect of OT on cognitive abilities. The increase in olfactory sensitivity was significantly related with an increase in odor discrimination (*r* = 0.45, *p* < 0.001), odor identification (*r* = 0.40, *p* < 0.001), and MoCA score (*r* = 0.33, *p* = 0.02). The increase of odors significance was accompanied with an increase in retronasal olfaction (*r* = 0.31, *p* = 0.03) and a decrease in cognitive performance (*r* = -0.31, *p* = 0.03). An increase in verbal associations as a function of OT was related with an increase in positive affect (*r* = 0.27, *p* = 0.05). Finally, the increase in negative affect after OT had positive relationship with an increase in depressive symptoms (*r* = 0.42, *p* < 0.001) and the decrease in positive affect (*r* = − 0.29, *p* = 0.03). All correlation coefficients are summarized in Table 2.

**Table 2:** Pearson’s correlations for the relationships between OT duration (interval), subjects’ age, and the change (Δ) in all measurements

	Age	OT duration	Δ Threshold	Δ Discrimination	Δ Identification	Δ Retronasal	Δ Individual significance of olfaction	Δ MoCA	Δ COWAT	Δ Verbal semantic fluency	Δ Depressive symptoms	Δ Positive affect
OT duration												
*r*	.14											
*p*	.30											
Δ Threshold												
*r*	.01	− .08										
*p*	.95	.56										
Δ Discrimination												
*r*	.05	− .16	.45^**^									
*p*	.73	.27	< .001									
Δ Identification												
*r*	− .17	− .20	.40^**^	.18								
*p*	.21	.16	< .001	.19								
Δ Retronasal												
*r*	− .25	− .13	.08	− .19	.02							
*p*	.07	.35	.56	.17	.87							
Δ Individual significance of olfaction												
*r*	− .06	.05	.03	.02	.02	.31^*^						
*p*	.67	.73	.85	.90	.87	.03						
Δ MoCA												
*r*	− .02	− .36^**^	.33^*^	.14	.04	.06	− .31^*^					
*p*	.86	.01	.02	.33	.80	.65	.03					
Δ COWAT												
*r*	− .01	.24	− .07	.07	− .19	.06	.22	− .18				
*p*	.95	.08	.62	.62	.17	.70	.11	.20				
Δ Verbal semantic fluency												
*r*	.05	− .16	.26	.20	.12	− .04	.21	.18	.11			
*p*	.71	.25	.06	.15	.39	.76	.14	.21	.43			
Δ Depressive symptoms												
*r*	.06	− .04	− .20	− .16	− .06	− .02	− .10	− .16	.12	.01		
*p*	.69	.76	.16	.25	.70	.89	.48	.26	.40	.93		
Δ Positive effect												
*r*	.29^*^	.24	− .01	.14	− .14	− .18	.04	.01	.27^*^	.15	− .20	
*p*	.03	.08	.96	.32	.32	.21	.76	.99	.05	.27	.16	
Δ Negative effect												
*r*	− .15	.13	.11	− .20	− .14	.18	− .07	− .15	− .01	.02	.42^**^	− .291^*^
*p*	.28	.37	.46	.17	.31	.20	.61	.27	.92	.91	< .001	.03

^***^ p < .001; ** *p* < .01; * *p* < .05

Subjects whose Sniffin’ Sticks score improved by at least 5.5 points or threshold score improved by 2.5 point or identification score improved by 3 points are considered to improve in clinical terms [36]. In our sample, significant improvement was independent from the cause of olfactory loss, yet the sample sizes for each group are small. The proportion of significantly proved patients clearly points to the patients with post-infectious olfactory loss as most responsive to OT treatment, whereas post-traumatic patients exhibited none-to-marginal improvement, as summarized in Table 3.

**Table 3:** The fraction of clinically significant improved patients with regard to the cause of olfactory loss

	Criterium		
	△TDI ≥ 5.5	△Thr ≥ 2.5	△Id ≥ 3
Controls	2 (6.9%)	6 (20.7%)	0 (0%)
Patients			
With idiopathic olfactory loss	1 (11.1%)	1 (11.1%)	1 (11.1%)
With post-infectious olfactory loss	3 (25%)	4 (33.3%)	2 (16.7%)
With post-traumatic olfactory loss	0 (0%)	1 (33.3%)	0 (0%)
*χ*^2^ (significance level)	3.19 (*p* = .36)	1.73 (*p* = .63)	5.14 (*p* = .16)

*TDI* combined Sniffin’ Sticks score for Threshold, Discrimination and Identification, *Thr* Threshold, *Id* Identification. Olfactory performance scores for two subjects (one with post-infectious and one with post-traumatic olfactory loss) could not be obtained during the second measurement (as mentioned in the section “Procedure”), and therefore, the improvement fraction was calculated for the total number of 12 patients with post-infectious olfactory loss and 3 subjects with post-traumatic olfactory loss

## Discussion

OT was equally beneficial for olfactory sensitivity of patients and control groups, suggesting that OT may be used not only to restore olfactory function in people diagnosed with olfactory impairment, but it may also be successfully used to enhance olfactory performance in subjects with normosmia. Standard OT regimen with two series of sniffs in the morning and in the evening turned out to be more effective than the intense OT with four series of sniffs. There are two plausible explanations of this outcome. First refers to the uneven allocation of patients with post-infectious olfactory loss to the OT regimen groups. Subjects who lost their sense of smell after an infection are most responsive to OT and if overrepresented in one of the experimental groups, could artificially create a statistical effect. In the case of our study, this explanation is rather unlikely due the insignificant post hoc *χ*^2^ tests for equal distribution. Yet, our sample size is small and could only reveal robust effects, whereas such effect could be very subtle. It is therefore recommended for future studies to carefully monitor allocation of subjects varying in the cause of olfactory loss to the experimental groups to avoid statistical artifacts.

The second plausible explanation of better effectiveness of the standard OT regimen than intense OT regimen refers to the lower baseline scores of the subjects in the standard OT regimen group. Despite the random assignment to the training regimens, baseline score for both olfactory sensitivity and verbal semantic fluency was lower for the standard OT regimen than the intense OT regimen. The better outcomes of OT in the group with lower baseline scores may suggest that the effectiveness of OT with regard to olfactory sensitivity and verbal semantic fluency is prone to a ceiling effect, and therefore, its effectiveness may be limited only to those subjects who presented relatively low scores at the baseline. From the motivational standpoint, improving from none-to-marginal odor perception at the baseline to some odor perception will be more noticeable and rewarding than improving within the range of hyposmic odor perception. On the other hand, the low baseline odor perception may favor leaving the study cohort. Subjects who resigned from the participation in our study were those with lower olfactory function at the first testing session—9 were patients and 1 was a control subject. Therefore, our study favors the notion about daily frustration with odors in subjects who can benefit from the OT most over the notion that the improvement capacity build subject’s motivation to perform the OT. The motivation to remain in the study cohort should be monitored in the future to better adjust procedure and prevent non-random drop outs.

Rehabilitation of olfactory system with the use of OT is most effective in patients with post-infectious olfactory loss (PIOL) [23, 37]. Our findings are in line with former studies showing greatest responsiveness to OT in PIOL patients [12]. The assumption that OT is a successful rehabilitation method of the olfactory loss caused by the infection is particularly important considering the global health crisis caused by the spread of SARS-CoV-2 known to attack the olfactory system.

OT yielded significant improvement of verbal semantic fluency in our subjects performing the training twice a day. This finding concurs former reports showing beneficial effects of OT on verbal function in older people (along with elevated well-being and olfactory performance) [38]. This cross-modal transfer of OT effects from olfaction to cognition also corroborates the initial reports on the olfactory-visual memory transfer as a result of OT [39]. Interestingly, although we did not observe significant effects of OT on cognitive function, other studies do suggest the relationship between olfactory perception and MoCA score [38]. The reason for the currently reported null-result may refer to the negative relationship between the between-measurements change in MoCA score and the duration of OT. Possibly, the effect of OT on cognitive assessment is transitory, but this requires further research. Alternatively, improvement in cognitive assessment may be dependent from OT compliance.

Unlike Wegener’s study [12], we did not observe significant effects of OT on depressive symptoms or affective state. We speculate that the lack of effects of OT on depressive symptoms may be related with the ceiling effect. In our sample, all subjects scored =  < 9 points, suggesting none-to-minimal depression [34]. Subjects with low BDI scores are more likely to comply with the OT regimen, whereas those with high BDI scores may be discouraged to perform training and expect negative results [40]. Finally, the null results of OT on BDI symptoms and positive/negative affect may not be discernible, because both questionnaires (BDI and PANAS) relate to a shorter time frame than the period of OT used in this study. Thus, future studies should use a shorter time frame to capture presumable effects of OT on emotional functioning.

In conclusion, results indicate that OT is effective in supporting olfactory rehabilitation and interventions targeted to verbal semantic fluency. However, it may be prone to a ceiling effect, being efficient in subjects presenting with lower baseline olfactory performance and lower verbal semantic fluency. Superiority of a more intense olfactory training could not be fully explained by this study and further research is needed to assess the potential benefit of more intense training regimes.

## Supplementary Information

Below is the link to the electronic supplementary material.

Supplementary file 1.

Supplementary file 2.

## Data Availability

The data that support the findings of this study are available from the corresponding author upon reasonable request.
