# Supplementary material for: Olfactory training with Aromastics: olfactory and cognitive effects
Source: Eur Arch Otorhinolaryngol. 2021 Apr 16;279(1):225–32. doi: 10.1007/s00405-021-06810-9 (PMC8051546; doi:10.1007/s00405-021-06810-9)
Supplement: Supplementary file 1 [file 405_2021_6810_MOESM1_ESM.docx]

*Table 1: Statistical coefficients for the models examining the effects of OT regimen, group and timepoint measurement with control of between-session interval and subjects’ age*

| Variable | Factor | Type III Sum of Squares | *df* | *F* | *p* |
| --- | --- | --- | --- | --- | --- |
| Threshold | Measurement | 0.34 | 1,47 | .11 | 0.737 |
|  | Regimen | 2.30 | 1,47 | .32 | 0.574 |
|  | Group | 704.10 | 1,47 | 98.20 | < .001 |
|  | Interval | 99.52 | 1,47 | 13.88 | .001 |
|  | Age | 5.33 | 1,47 | .74 | .393 |
|  | Measurement * Age | 0.15 | 1,47 | .05 | .823 |
|  | Measurement * Interval | 1.16 | 1,47 | .39 | .535 |
|  | Measurement * Regimen | 17.82 | 1,47 | 5.97 | .018 |
|  | Measurement* Group | 3.34 | 1,47 | 1.12 | .295 |
|  | Regimen * Group | 0.34 | 1,47 | .05 | .83 |
|  | Measurement * Regimen * Group | 2.45 | 1,47 | .82 | .369 |
| Discrimination | Measurement | 0.92 | 1,47 | .24 | .629 |
|  | Regimen | 2.23 | 1,47 | .21 | .65 |
|  | Group | 313.07 | 1,47 | 29.32 | < .001 |
|  | Interval | 18.13 | 1,47 | 1.67 | .199 |
|  | Age | 15.66 | 1,47 | 1.45 | .232 |
|  | Measurement * Age | 2.14 | 1,47 | .55 | .463 |
|  | Measurement * Interval | 13.11 | 1,47 | 3.36 | .073 |
|  | Measurement * Regimen | 1.41 | 1,47 | .36 | .551 |
|  | Measurement* Group | <.01 | 1,47 | <.01 | .992 |
|  | Regimen * Group | 0.76 | 1,47 | .07 | .79 |
|  | Measurement * Regimen * Group | 18.83 | 1,47 | 4.83 | .033^a^ |
| Identification | Measurement | 4.17 | 1,47 | 2.31 | .135 |
|  | Regimen | 17.96 | 1,47 | 1.48 | .229 |
|  | Group | 617.50 | 1,47 | 51.03 | < .001 |
|  | Interval | 35.98 | 1,47 | 2.97 | .091 |
|  | Age | 2.75 | 1,47 | .23 | .636 |
|  | Measurement * Age | 2.82 | 1,47 | 1.56 | .218 |
|  | Measurement * Interval | 0.84 | 1,47 | .47 | .499 |
|  | Measurement * Regimen | 0.01 | 1,47 | .01 | .933 |
|  | Measurement* Group | 2.40 | 1,47 | 1.33 | .255 |
|  | Regimen * Group | 9.66 | 1,47 | .80 | .376 |
|  | Measurement * Regimen * Group | 0.58 | 1,47 | .32 | .574 |
| Retronasal olfaction | Measurement | 4.85 | 1,47 | 3.12 | .084 |
|  | Regimen | 0.71 | 1,47 | .07 | .799 |
|  | Group | 324.33 | 1,47 | 29.97 | < .001 |
|  | Interval | 33.85 | 1,47 | 3.13 | .083 |
|  | Age | 2.68 | 1,47 | .243 | .621 |
|  | Measurement * Age | 5.63 | 1,47 | 3.63 | .063 |
|  | Measurement * Interval | 0.02 | 1,47 | .01 | .917 |
|  | Measurement * Regimen | 5.79 | 1,47 | 3.73 | .059 |
|  | Measurement* Group | 0.03 | 1,47 | .02 | .898 |
|  | Regimen * Group | 34.17 | 1,47 | 3.16 | .082 |
|  | Measurement * Regimen * Group | 4.40 | 1,47 | 2.8 | .099 |
| Olfaction importance | Measurement | 1.08 | 1,47 | .03 | .873 |
|  | Regimen | 232.99 | 1,47 | 1.81 | .185 |
|  | Group | 209.64 | 1,47 | 1.62 | .209 |
|  | Interval | 0.63 | 1,47 | <.01 | .945 |
|  | Age | 286.82 | 1,47 | 2.22 | .143 |
|  | Measurement * Age | 2.10 | 1,47 | .05 | .824 |
|  | Measurement * Interval | 1.02 | 1,47 | .02 | .877 |
|  | Measurement * Regimen | 40.79 | 1,47 | .98 | .328 |
|  | Measurement* Group | 138.78 | 1,47 | 3.32 | .075 |
|  | Regimen * Group | 320.43 | 1,47 | 2.48 | .122 |
|  | Measurement * Regimen * Group | 2.57 | 1,47 | .06 | .805 |
| B_F_L | Measurement | 2.66 | 1,48 | 0.15 | .701 |
|  | Regimen | 723.84 | 1,48 | 5.44 | .024 |
|  | Group | 357.84 | 1,48 | 2.67 | .108 |
|  | Interval | 7.79 | 1,48 | .06 | .810 |
|  | Age | 66.54 | 1,48 | .50 | .483 |
|  | Measurement * Age | 1.72 | 1,48 | .10 | .758 |
|  | Measurement * Interval | 49.63 | 1,48 | 2.77 | .102 |
|  | Measurement * Regimen | 2.22 | 1,48 | .12 | .726 |
|  | Measurement* Group | <.01 | 1,48 | <.01 | .994 |
|  | Regimen * Group | 45.87 | 1,48 | .34 | .560 |
|  | Measurement * Regimen * Group | 0.29 | 1,48 | .02 | .899 |
| Supermarket | Measurement | 4.07 | 1,48 | .58 | .449 |
|  | Regimen | 221.96 | 1,48 | 20.26 | < .001 |
|  | Group | 78.29 | 1,48 | 7.14 | .010 |
|  | Interval | 8.50 | 1,48 | .78 | .383 |
|  | Age | 0.36 | 1,48 | .03 | .856 |
|  | Measurement * Age | 3.93 | 1,48 | .56 | .457 |
|  | Measurement * Interval | 6.88 | 1,48 | .99 | .326 |
|  | Measurement * Regimen | 128.42 | 1,48 | 18.40 | < .001 |
|  | Measurement* Group | 2.45 | 1,48 | .35 | .556 |
|  | Regimen * Group | 5.19 | 1,48 | .47 | .494 |
|  | Measurement * Regimen * Group | 0.02 | 1,48 | <.01 | .960 |
| MoCA | Measurement | 2.63 | 1,48 | 3.73 | .059 |
|  | Regimen | 0.23 | 1,48 | .13 | .718 |
|  | Group | 14.75 | 1,48 | 8.49 | .005 |
|  | Interval | 0.06 | 1,48 | .04 | .849 |
|  | Age | 13.46 | 1,48 | 7.753 | .008 |
|  | Measurement * Age | 0.01 | 1,48 | .01 | .925 |
|  | Measurement * Interval | 3.96 | 1,48 | 5.60 | .022 |
|  | Measurement * Regimen | 0.56 | 1,48 | .79 | .379 |
|  | Measurement* Group | 2.66 | 1,48 | 3.76 | .058 |
|  | Regimen * Group | 0.52 | 1,48 | .30 | .585 |
|  | Measurement * Regimen * Group | 0.38 | 1,48 | .53 | .469 |
| BDI | Measurement | 0.04 | 1,46 | .06 | .808 |
|  | Regimen | 13.38 | 1,46 | 2.18 | .147 |
|  | Group | 5.04 | 1,46 | .82 | .370 |
|  | Interval | 6.39 | 1,46 | 1.04 | .313 |
|  | Age | 3.78 | 1,46 | .61 | .437 |
|  | Measurement * Age | 0.10 | 1,46 | .14 | .706 |
|  | Measurement * Interval | <.01 | 1,46 | .01 | .939 |
|  | Measurement * Regimen | 0.02 | 1,46 | .03 | .864 |
|  | Measurement* Group | 0.26 | 1,46 | .36 | .549 |
|  | Regimen * Group | 0.61 | 1,46 | .10 | .755 |
|  | Measurement * Regimen * Group | 0.61 | 1,46 | .85 | .362 |
| PANAS Positive | Measurement | 0.35 | 1,47 | 6.45 | .014 |
|  | Regimen | 0.10 | 1,47 | .18 | .674 |
|  | Group | 0.03 | 1,47 | .06 | .814 |
|  | Interval | 0.34 | 1,47 | .60 | .443 |
|  | Age | 0.77 | 1,47 | 1.36 | .249 |
|  | Measurement * Age | 0.17 | 1,47 | 3.14 | .083 |
|  | Measurement * Interval | 0.17 | 1,47 | 3.06 | .087 |
|  | Measurement * Regimen | 0.01 | 1,47 | .11 | .746 |
|  | Measurement* Group | <.01 | 1,47 | .02 | .891 |
|  | Regimen * Group | 1.21 | 1,47 | 2.16 | .149 |
|  | Measurement * Regimen * Group | 0.04 | 1,47 | .81 | .373 |
| PANAS Negative | Measurement | 0.03 | 1,47 | .02 | .902 |
|  | Regimen | 3.82 | 1,47 | 1.78 | .189 |
|  | Group | 3.77 | 1,47 | 1.75 | .192 |
|  | Interval | 1.39 | 1,47 | .65 | .425 |
|  | Age | 0.80 | 1,47 | .37 | .546 |
|  | Measurement * Age | 3.67 | 1,47 | 1.78 | .188 |
|  | Measurement * Interval | 4.90 | 1,47 | 2.38 | .130 |
|  | Measurement * Regimen | 3.69 | 1,47 | 1.79 | .188 |
|  | Measurement* Group | 1.20 | 1,47 | .58 | .449 |
|  | Regimen * Group | 3.68 | 1,47 | 1.71 | .197 |
|  | Measurement * Regimen * Group | 6.28 | 1,47 | 3.05 | .087 |

^a^ – none of the planned pairwise comparisons were significant
