# Supplementary material for: Olfactory training with Aromastics: olfactory and cognitive effects
Source: Eur Arch Otorhinolaryngol. 2021 Apr 16;279(1):225–32. doi: 10.1007/s00405-021-06810-9 (PMC8051546; doi:10.1007/s00405-021-06810-9)
Supplement: Supplementary file 2 [file 405_2021_6810_MOESM2_ESM.docx]

*Table 2: Estimated marginal means for the models examining the effects of OT regimen, group and timepoint measurement with control of between-session interval and subjects’ age*

| Variable |  |  |  | Mean | Standard Error | 95% CI | |
| --- | --- | --- | --- | --- | --- | --- | --- |
|  |  |  |  |  |  | Low. Bound | Up. Bound |
| Threshold | Standard Regimen | Patients | Pre-training | 1.97 | .54 | .88 | 3.06 |
|  |  |  | Post-training | 3.19 | .71 | 1.76 | 4.63 |
|  |  | Control group | Pre-training | 7.55 | .47 | 6.60 | 8.51 |
|  |  |  | Post-training | 8.69 | .62 | 7.43 | 9.94 |
|  | Intense Regimen | Patients | Pre-training | 2.91 | .61 | 1.16 | 4.14 |
|  |  |  | Post-training | 3.10 | .80 | 1.49 | 4.72 |
|  |  | Control group | Pre-training | 8.90 | .58 | 7.73 | 10.08 |
|  |  |  | Post-training | 7.68 | .77 | 6.16 | 9.24 |
| Discrimination | Standard Regimen | Patients | Pre-training | 9.58 | .75 | 8.07 | 11.10 |
|  |  |  | Post-training | 8.56 | .76 | 7.03 | 10.10 |
|  |  | Control group | Pre-training | 12.47 | .66 | 11.43 | 13.79 |
|  |  |  | Post-training | 13.27 | .67 | 11.93 | 14.62 |
|  | Intense Regimen | Patients | Pre-training | 8.32 | .85 | 6.62 | 10.03 |
|  |  |  | Post-training | 9.60 | .86 | 7.86 | 11.33 |
|  |  | Control group | Pre-training | 12.66 | .81 | 11.03 | 14.28 |
|  |  |  | Post-training | 12.12 | .82 | 10.46 | 13.77 |
| Identification | Standard Regimen | Patients | Pre-training | 8.46 | .61 | 13.42 | 15.89 |
|  |  |  | Post-training | 8.95 | .78 | 7.39 | 10.52 |
|  |  | Control group | Pre-training | 14.66 | .61 | 13.42 | 15.89 |
|  |  |  | Post-training | 14.20 | .68 | 12.86 | 15.57 |
|  | Intense Regimen | Patients | Pre-training | 8.45 | .79 | 6.85 | 10.40 |
|  |  |  | Post-training | 8.58 | .88 | 6.82 | 10.34 |
|  |  | Control group | Pre-training | 13.02 | .76 | 11.51 | 14.54 |
|  |  |  | Post-training | 12.84 | .84 | 11.16 | 14.52 |
| Retronasal olfaction | Standard Regimen | Patients | Pre-training | 12.95 | .66 | 11.61 | 14.28 |
|  |  |  | Post-training | 13.90 | .73 | 12.43 | 15.38 |
|  |  | Control group | Pre-training | 18.32 | .58 | 17.16 | 19.49 |
|  |  |  | Post-training | 18.33 | .64 | 17.05 | 19.62 |
|  | Intense Regimen | Patients | Pre-training | 15.26 | .75 | 13.76 | 16.76 |
|  |  |  | Post-training | 14.38 | .83 | 12.72 | 16.04 |
|  |  | Control group | Pre-training | 17.30 | .71 | 15.87 | 18.74 |
|  |  |  | Post-training | 17.24 | .79 | 15.65 | 18.82 |
| Olfaction Importance | Standard Regimen | Patients | Pre-training | 34.73 | 2.69 | 29.33 | 40.14 |
|  |  |  | Post-training | 34.35 | 3.00 | 28.31 | 40.38 |
|  |  | Control group | Pre-training | 31.81 | 2.13 | 27.52 | 36.10 |
|  |  |  | Post-training | 35.51 | 2.38 | 30.73 | 40.30 |
|  | Intense Regimen | Patients | Pre-training | 29.50 | 2.54 | 24.39 | 34.60 |
|  |  |  | Post-training | 25.88 | 2.83 | 20.19 | 31.58 |
|  |  | Control group | Pre-training | 33.52 | 2.64 | 28.21 | 29.82 |
|  |  |  | Post-training | 35.36 | 2.94 | 29.44 | 41.28 |
| COWAT | Standard Regimen | Patients | Pre-training | 32.87 | 2.32 | 28.20 | 37.54 |
|  |  |  | Post-training | 27.67 | 2.77 | 33.12 | 44.23 |
|  |  | Control group | Pre-training | 38.18 | 1.93 | 34.31 | 42.06 |
|  |  |  | Post-training | 43.75 | 2.30 | 39.14 | 48.37 |
|  | Intense Regimen | Patients | Pre-training | 39.99 | 2.30 | 35.36 | 44.61 |
|  |  |  | Post-training | 44.98 | 2.74 | 39.48 | 50.49 |
|  |  | Control group | Pre-training | 42.24 | 2.39 | 37.43 | 47.04 |
|  |  |  | Post-training | 47.45 | 2.84 | 41.73 | 53.16 |
| Verbal semantic fluency | Standard Regimen | Patients | Pre-training | 17.84 | 1.10 | 15.64 | 20.05 |
|  |  |  | Post-training | 22.84 | .59 | 21.66 | 24.03 |
|  |  | Control group | Pre-training | 20.37 | .91 | 18.54 | 22.20 |
|  |  |  | Post-training | 24.80 | .49 | 23.82 | 25.79 |
|  | Intense Regimen | Patients | Pre-training | 23.45 | 1.09 | 21.27 | 25.64 |
|  |  |  | Post-training | 24.05 | .59 | 22.87 | 25.23 |
|  |  | Control group | Pre-training | 25.08 | 1.13 | 22.82 | 27.35 |
|  |  |  | Post-training | 25.00 | .61 | 23.78 | 26.22 |
| MoCA | Standard Regimen | Patients | Pre-training | 27.80 | .37 | 27.06 | 28.54 |
|  |  |  | Post-training | 28.69 | .27 | 28.13 | 29.24 |
|  |  | Control group | Pre-training | 28.91 | .31 | 28.30 | 29.53 |
|  |  |  | Post-training | 29.41 | .23 | 28.95 | 29.86 |
|  | Intense Regimen | Patients | Pre-training | 28.06 | .37 | 27.33 | 28.80 |
|  |  |  | Post-training | 28.91 | .27 | 28.37 | 29.46 |
|  |  | Control group | Pre-training | 29.13 | .38 | 28.37 | 29.89 |
|  |  |  | Post-training | 29.07 | .28 | 28.51 | 29.64 |
| BDI | Standard Regimen | Patients | Pre-training | 1.15 | .55 | .04 | 2.27 |
|  |  |  | Post-training | 1.30 | .59 | .12 | 2.48 |
|  |  | Control group | Pre-training | .59 | .44 | -.30 | 1.47 |
|  |  |  | Post-training | .61 | .47 | -.34 | 1.55 |
|  | Intense Regimen | Patients | Pre-training | 1.92 | .52 | .87 | 2.97 |
|  |  |  | Post-training | 1.67 | .56 | .55 | 2.79 |
|  |  | Control group | Pre-training | 1.36 | .57 | .21 | 2.50 |
|  |  |  | Post-training | 1.65 | .61 | .43 | 2.87 |
| PANAS Positive | Standard Regimen | Patients | Pre-training | 3.35 | .17 | 3.00 | 3.69 |
|  |  |  | Post-training | 3.45 | .17 | 3.10 | 3.79 |
|  |  | Control group | Pre-training | 3.67 | .14 | 3.39 | 3.94 |
|  |  |  | Post-training | 3.67 | .14 | 3.39 | 3.94 |
|  | Intense Regimen | Patients | Pre-training | 3.58 | .16 | 3.25 | 3.890 |
|  |  |  | Post-training | 3.56 | .16 | 3.23 | 3.88 |
|  |  | Control group | Pre-training | 3.34 | .17 | 3.00 | 3.68 |
|  |  |  | Post-training | 3.40 | .17 | 3.06 | 3.74 |
| PANAS Negative | Standard Regimen | Patients | Pre-training | 1.38 | .61 | .14 | 2.61 |
|  |  |  | Post-training | 1.82 | .166 | 1.50 | 2.13 |
|  |  | Control group | Pre-training | 1.70 | .49 | .73 | 2.68 |
|  |  |  | Post-training | 1.52 | .12 | 1.27 | 1.77 |
|  | Intense Regimen | Patients | Pre-training | 3.09 | .58 | 1.93 | 4.25 |
|  |  |  | Post-training | 1.70 | .15 | 1.40 | 1.99 |
|  |  | Control group | Pre-training | 1.53 | .60 | .32 | 2.74 |
|  |  |  | Post-training | 1.65 | .15 | 1.35 | 1.96 |
